# Comparison between Variable-Selection Algorithms in PLS Regression with Near-Infrared Spectroscopy to Predict Selected Metals in Soil

**DOI:** 10.3390/molecules28196959

**Published:** 2023-10-06

**Authors:** Giovanna Abrantes, Valber Almeida, Angelo Jamil Maia, Rennan Nascimento, Clistenes Nascimento, Ygor Silva, Yuri Silva, Germano Veras

**Affiliations:** 1 Departamento de Química, Centro de Ciência e Tecnologia, Universidade Estadual da Paraíba, Campina Grande 58429-500, Brazil; giovanna.fatima.abrantes@aluno.uepb.edu.br (G.A.); vallber_ellias@hotmail.com (V.A.); 2 Agronomy Department, Federal Rural University of Pernambuco, Recife 52171-900, Brazil; request.angelo@gmail.com (A.J.M.); rennancabral2@yahoo.com.br (R.N.); cwanascimento@hotmail.com (C.N.); ygor.silva@ufrpe.br (Y.S.); 3 Agronomy Department, Federal University of Piauí, Bom Jesus 64900-000, Brazil; yurijacques@ufpi.edu.br

**Keywords:** metal content, vibrational spectroscopy, chemometrics, FFiPLS, multivariate calibration

## Abstract

Soil is one of the Earth’s most important natural resources. The presence of metals can decrease environmental quality if present in excessive amounts. Analyzing soil metal contents can be costly and time consuming, but near-infrared (NIR) spectroscopy coupled with chemometric tools can offer an alternative. The most important multivariate calibration method to predict concentrations or physical, chemical or physicochemical properties as a chemometric tool is partial least-squares (PLS) regression. However, a large number of irrelevant variables may cause problems of accuracy in the predictive chemometric models. Thus, stochastic variable-selection techniques, such as the Firefly algorithm by intervals in PLS (FFiPLS), can provide better solutions for specific problems. This study aimed to evaluate the performance of FFiPLS against deterministic PLS algorithms for the prediction of metals in river basin soils. The samples had their spectra collected from the region of 1000–2500 nm. Predictive models were then built from the spectral data, including PLS, interval-PLS (iPLS), successive projections algorithm for interval selection in PLS (iSPA-PLS), and FFiPLS. The chemometric models were built with raw data and preprocessed data by using different methods such as multiplicative scatter correction (MSC), standard normal variate (SNV), mean centering, adjustment of baseline and smoothing by the Savitzky–Golay method. The elliptical joint confidence region (EJCR) used in each chemometric model presented adequate fit. FFiPLS models of iron and titanium obtained a relative prediction deviation (RPD) of more than 2. The chemometric models for determination of aluminum obtained an RPD of more than 2 in the preprocessed data with SNV, MSC and baseline (offset + linear) and with raw data. The metals Be, Gd and Y failed to obtain adequate models in terms of residual prediction deviation (RPD). These results are associated with the low values of metals in the samples. Considering the complexity of the samples, the relative error of prediction (REP) obtained between 10 and 25% of the values adequate for this type of sample. Root mean square error of calibration and prediction (RMSEC and RMSEP, respectively) presented the same profile as the other quality parameters. The FFiPLS algorithm outperformed deterministic algorithms in the construction of models estimating the content of Al, Be, Gd and Y. This study produced chemometric models with variable selection able to determine metals in the Ipojuca River watershed soils using reflectance-mode NIR spectrometry.

## 1. Introduction

Soil is unique due to the many different living systems of chemical species it incorporates. An environmental diagnosis has listed some of the following problems that affect soil, the remaining vegetation cover, and the surface and groundwater in a river basin: deforestation, advancement of agricultural activity, exposed soils on slopes used for clay, gravel and sand extraction on river banks, disorderly occupation, discharge of domestic and industrial effluents, and disposal of solid waste. In this sense, soil conditions determine the nature of plant ecosystems and the ability of the land to support life, and indicate the presence of contaminants, whether they originate from natural or human-made sources due to population growth, urbanization and poor management [1].

The toxicity caused by inorganic contaminants in soil is considered higher than that caused by combined organic and radioactive sources [2]. Beryllium, for example, present in alloys is used in aerospace, electronics and mechanical industries, but its compounds are carcinogenic, immune system suppressers, cell division reducers and genotoxic to animals and humans [3]. Yttrium and gadolinium, in turn, are rare-earth elements employed in high-tech production and clean energy products and economic exploitation, and they have gained worldwide interest [4,5]. These elements, however, affect the human health in the digestive, respiratory, reproductive, neurological, hematological and immune systems [6,7]. Titanium is a heavy metal applied in metal alloys with few risks to human health but with possible harmful effects still being studied [8]. Aluminum may cause nausea, mouth and skin ulcers and arthritic pain, and increases the risk of Alzheimer’s disease, among other problems [9]. On the other hand, iron is an essential component in the cells of all living organisms and its utility is well known. The last two cited metals, Al and Fe, are used in many transformation industries with direct implications for economic, social and technological development.

The most common methods used for the determination of the aforementioned analytes in soil consist of the application of inductively coupled plasma optical emission spectrometry (ICP-OES), atomic absorption spectrometry (AAS) and X-ray diffraction (XRD) [10,11,12,13]. These methods are expensive due to the quantity of steps during the extraction process and the high consumption of reagents and instrumentation. As a viable alternative, some studies using reflectance spectroscopy in the near-infrared region for prediction of some soil properties have been developed [14,15,16,17,18,19].

Krzebietke et al. [14] used NIR spectroscopy to determine metals in cultivated Haplic Luvisol soils in Balcyny near Ostroda, Poland. The proposed method was applied to determine very low concentrations of Cd, Cu, Ni and Cr and high concentrations of Zn, Mn and Fe. The authors point out that the results were adequate to determine all studied metals using the coefficients of determination as the quality parameter for the model.

Fonseca et al. [15] developed a protocol to guarantee the representativity of this measurement in the determination of organic carbon in Clay Ferrasol Soil in São Sebastião da Vargem Grande, Mina Gerais State, Brazil. Four measurement models were studied; the best result, using almost all wavelengths of the NIR spectrum, provided information for SOC determination and presented high stability during the calibration process in the NIR spectrophotometer.

Haghi et al. [16] predicted various soil properties comparing NIR with Fourier-transform infrared (FTIR) spectroscopies. The spectroscopic dataset used in this work was extracted from the National Soil Inventory of Scotland. The properties evaluated were total carbon, total nitrogen, bulk density, clay, sand, silt, pH (in H_2_O), exchangeable Mg and exchangeable K. The authors concluded that the overall performance to determine the parameters of FTIR under study, except for pH, was better than the NIR spectroscopy.

Jia et al. [17] developed a method to determine soil nitrogen and organic carbon. The samples were obtained in nine towns in Wencheng county, Zhejiang province, China. The authors concluded that the residual prediction deviations were adequate for both parameters using NIR spectroscopy.

Oliveira et al. [18] proposed a method to determine sand, silt and clay in high concentrations and Th and total rare-earth elements in low quantities. The soil profiles were located in Borborema Province, Pernambuco State, northeastern Brazil. The authors concluded that the models constructed were adequate to determine the studied parameters using NIR spectroscopy.

Maia et al. [19] used NIR spectroscopy to determine metals in soil and sediment samples obtained in the Ipojuca river basin in the state of Pernambuco, northeastern Brazil. The authors concluded that for the prediction of Co, Cr, Mo, Ni and Sn, this method presented a poor performance. Satisfactory results were achieved for Al, Ti, Sc and V, and reasonable results were achieved for Fe, La, Mn, Pr, Sm, Sr and Th.

The articles cited above used NIR spectroscopy to determine properties or concentration of metals in soils, treating the data using chemometric tools. This treatment was associated with the NIR spectra’s broad and overlapping overtones and combination bands, i.e., a great deal of information in a short spectral region with low signal. In this context, the use of chemometric tools is necessary to describe the relationship between spectra signal and quantity of interest.

Multivariate calibration is a process that associates the concentration of a given analyte/property with a measured response that can come from such things as near-infrared spectra and chromatographic profiles [20,21,22]. The partial least-squares regression (PLSR) algorithm is among the deterministic methods that have stood out in the last thirty years for their versatility [23]. This method is regarded as an excellent regression algorithm because it is efficient even in the presence of non-explicative variables.

Conceptually, PLSR reduces the influence of uninformative or noisy variables by applying low weights to these variables in the models constructed. Despite this, variable-selection strategies can still be used to reduce dimensionality in a large dataset, minimizing redundancy and excluding uninformative or noisy variables. Variable-selection techniques are widely applied to improve the performance of chemometric PLSR models in terms of the figures of merit, such as accuracy and precision, mainly when using a small number of samples [17,24,25].

Two types of procedures can be employed: deterministic and stochastic algorithms [26,27,28,29]. In the case of specific optimization problems with high dimensionality, stochastic algorithms are widely employed because they seek better solutions involving randomness, such as bio-inspired algorithms [18,30].

Among the bio-inspired algorithms, our research group developed the Firefly algorithm by intervals in PLS (FFiPLS) [18]. This algorithm is based on the bioluminescence behavior of fireflies when searching for food. In this procedure, one or more variable intervals may be chosen to improve the quality of a PLS model.

In view of the above, this study aims to evaluate the performance of FFiPLS against deterministic algorithms such as iPLS, iSPA-PLS and full PLSR from raw and preprocessed soil using NIR spectra to build models for the prediction of aluminum, beryllium, iron, titanium, gadolinium and yttrium content in soil. These metals were chosen based on their presence in the samples and important uses in industries and technological products. Iron, aluminum and titanium were used due to their high quantity in the soil samples. In all cases, NIR was able to resolve some problems with the reference analytical techniques. 

## 2. Results

Table 1 presents basic statistics regarding the determination of selected metals (Al, Be, Fe, Ti, Gd and Y) in soil samples. Among these analytes, there were higher concentrations of aluminum (Al), iron (Fe) and titanium (Ti). This can be attributed to their greater abundance in the Earth’s crust. Al is the most abundant metal in the crust, constituting around 8% of its composition, closely followed by Fe, which comprises approximately 5% [31]. Additionally, Ti, though less abundant than Al and Fe, still occurs in significant amounts. On the other hand, beryllium (Be), gadolinium (Gd) and yttrium (Y) are much less abundant in the Earth’s crust. Be is commonly described as a trace metal [32], while Gd and Y are both rare-earth elements [33], present in average to low concentrations in soil. The RSD presented in Table 1 indicates a large range of metal concentrations able to build the chemometric models. The concentrations of Ti, Fe and Al were high, being the major components. The concentrations of Be, Gd and Y were lower. In terms of metals with low concentrations, the chemometric models were less reliable for the higher concentrations. 

**Figure 1:**
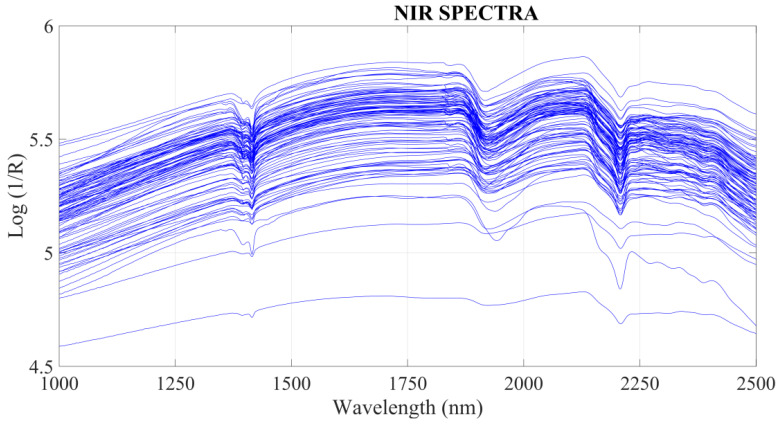
NIR spectra of soil samples.

The spectra were used for building the chemometric predictive models, and the data are presented in Figure 1. In terms of spectral profile, four samples had lower signal intensities than the others in some spectral regions. But this difference did not affect the results since the spectral profile was the same, differing only in the intensity of the bands.

Two small reflectance peaks were observed at 1450 and 1950 nm regions associated with vibrational frequencies of -OH groups arising from the adsorbed water. Furthermore, clay minerals were absorbed in the NIR due to combinations of metals with O-H and C-O stretching. Reflectance close to 2204 nm can be given due to combinations of Al-OH vibrations and 2280 nm by Fe-OH [34,35].

Depending on the wavelength, various soil properties can be detected directly. For the determination of metals, however, the relationship between the reflected energy in the near-infrared region (1000–2500 nm) is associated with the part of the organic coordination compound that produced an interaction pattern related to the vibrations caused by the elongation and bending of molecular bonds of clay, oxides and others.

The results of the chemometric models were available initially by the elliptical joint confidence region (EJCR) of calibration and prediction. These graphs must include the theoretical ideal point; for this, the models did not present significant bias. After the EJCR was obtained, the following other figures of merit were evaluated: latent variables, root mean square error of cross validation (RMSECV), root mean square error of prediction (RMSEP), bias of prediction (bias_pred_), standard deviation of validation (SDV), ratio of performance to deviation (RPD) and relative error of prediction (REP) were available.

The choice of latent variables was given in the function of the smallest RMSECV. The models were built from the suggested latent variables by the algorithms. These results, however, were not promising compared to those determined by evaluating the smallest residual error.

### 2.1. Determination of Titanium

The EJCR of chemometric models (Table 2) proved to be adequate with lower latent variables for the preprocessed data when used with the FFiPLS algorithm from different preprocessing techniques (MSC, SNV and baseline fit). Models that showed overfitting were excluded, based on the high number of latent variables that added irrelevant information to the built models. The best model for Ti used 750 spectral variables with MSC as preprocessing in the FFiPLS algorithm with R^2^_cal_ equal to 0.8381, 0.92 × 10^3^ mg kg^−1^ of RMSEC, lower REP (15.60%) and RPD (2.16), and higher SDV (0.79 × 10^3^ mg kg^−1^) when employing 10 latent variables.

**Figure 2:**
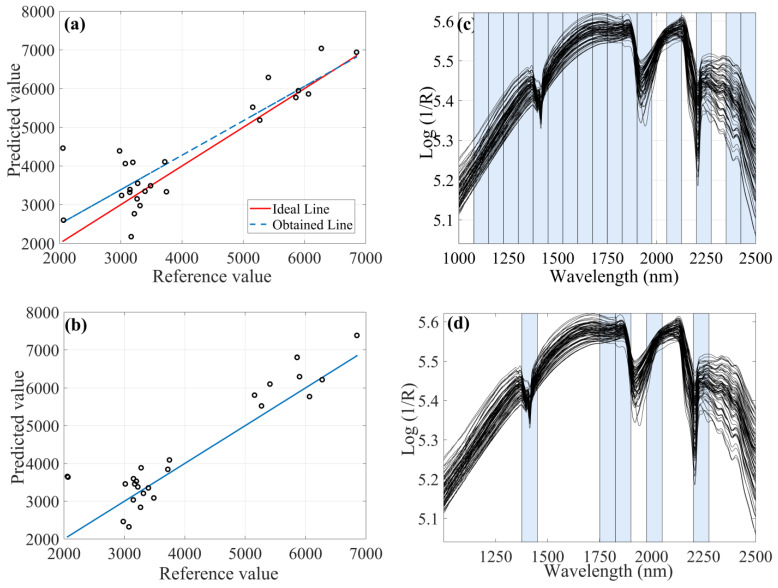
Chemometric models with MSC as preprocessing for determination of titanium content: (**a**) Prediction set by iSPA-PLS; (**b**) Prediction sample set by FFiPLS; (**c**) Selected spectral regions by iSPA-PLS; (**d**) Selected spectral regions by FFiPLS.

It should be noted that the deterministic algorithms showed possible overfitting when compared to the stochastic algorithm. Parameter calibration leads to the risk of overfitting. This usually occurs due to the choice of the appropriate set of instances during computational experimentation with a reasonable measure of difficulty and with a wide range of size. It was possible to observe, for example, that the iSPA-PLS algorithm using MSC preprocessing (Figure 2a) forced the result near to ideal using almost the full spectra but with 21 latent variables. The FFiPLS model obtained a similar result using the same preprocessing but with fewer latent variables.

The statistical significance between the RMSEP values was evaluated using the F-test to compare the reliability of the models, showing no statistically significant differences between them.

Titanium oxides may be related to average clay grain size composition with predominance of kaolinitic mineralogy and oxides. The FFiPLS model preprocessed by MSC used the spectral range 1375–1450 nm associated with vibrational frequencies of the hydroxyl radical (O-H) present in the water adsorbed by the vibrational combination of metal-hydroxyl plus O-H stretch in a 1:1 mineral structure. The spectral region 1600–1675 nm may be associated with vibrations of the oxygen bonds, confirming the adequate result of the cited chemometric model.

Maia et al. [19] determined titanium and other metals in soil using NIR spectrometry. The best chemometric model that was obtained used random forest as the calibration method and SNV as the preprocessing algorithm. In comparison to Maia et al., the proposed model in our article, using FFiPLS with MSC as the preprocessing data algorithm, obtained better RMSEP (0.62 × 10^3^ versus 0.93 × 10^3^ mg/kg), RPD (2.16 versus 2.02) and R^2^ (0.78 versus 0.74) using only 750 versus 2500 variables [19].

Tepanosyan et al. [36] proposed a method to determine Ti using NIR spectroscopy with PLS regression. The result was a better chemometric model with better RMSEP (0.33 × 10^3^ versus 0.62 × 10^3^ mg/kg) but worse R^2^ (0.71 versus 0.78) and higher latent variables (14 versus 10). They used two spectral regions with 300 wavelengths [36] in comparison to the proposed method in our study.

Naibo et al. [37] analyzed many metals, including Ti, with NIR spectroscopy with PLS regression. The best result obtained was a RMSEP of 0.11 × 10^3^ mg/kg using full spectra with the Savitzky–Golay derivative as the preprocessing method in NIR data but with an R^2^ equal to 0.99, which indicates an overfitting method. The authors indicated that this method of Ti determination was not accurate.

### 2.2. Determination of Iron

For iron, the model employing the FFiPLS algorithm with moving average preprocessing (Table 3) did not prove suitable due to the use of a larger number of latent variables (LVs = 16). In addition, the model produced higher RMSEP (8.09 × 10^3^ mg kg^−1^), bias (1.70 × 10^3^ mg kg^−1^) and SDV (8.79 × 10^3^ mg kg^−1^), with a high variance and a lower coefficient of determination for the prediction set.

The lowest bias_pred_ obtained for Fe was through the FFiPLS algorithm preprocessed by SNV (0.46 × 10^3^ mg kg^−1^). The deterministic iPLS algorithm preprocessed by SNV also proved to be interesting for the coefficients of determination and bias. FFiPLS used a smaller number of LVs for building the models cited in this study. In the literature, high iron content can be correlated with the low reflectance in the iron-oxide (Fe_2_O_3_) bands [38,39].

The results obtained showed high values of RMSECV, RMSEC, RMSEP, bias_pred_ and SDV but within the concentration range of the samples used (0.9–68.9 × 10^3^ mg kg^−1^). The FFiPLS model preprocessed by SNV showed higher RPDpred and better fit in terms of the prediction set, making it important to evaluate not only the coefficients of determination and RMSEs but also the whole set of figures of merit.

**Figure 3:**
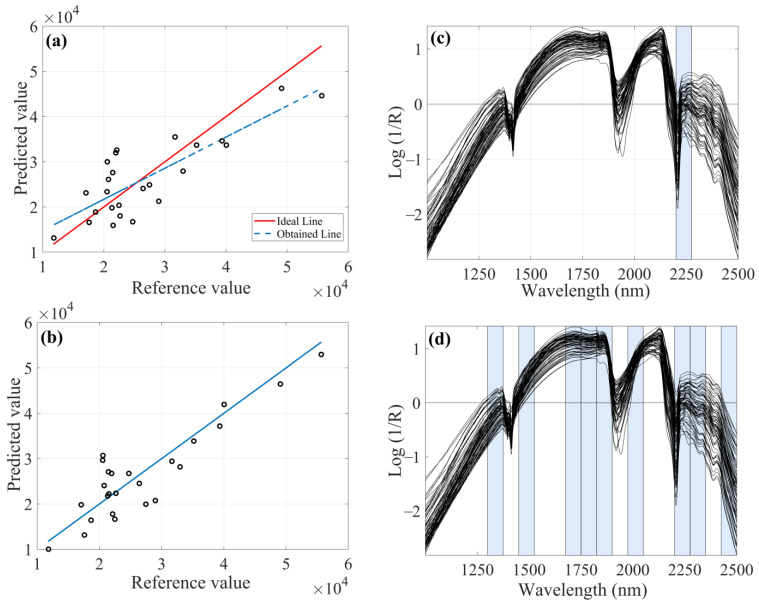
Chemometric models with SNV as preprocessing for determination of iron content: (**a**) Prediction sample set by iPLS; (**b**) Prediction sample set by FFiPLS; (**c**) Selected spectral regions by iPLS; (**d**) Selected spectral regions by FFiPLS.

Both SNV preprocessed models, iPLS and FFiPLS (Figure 3), selected the spectral range of 2200–2275 nm. Iron in soil can be associated with complexes, such as adsorbed organic matter. Cations such as Fe^3+^ can be attracted to low-molecular-mass organic acids at the edges of mineral structures, which chelate or bind them into stable organometallic complexes. An absorption near to 2280 nm may be associated with the presence of iron hydroxides with Fe replaced in the octahedral form. Iron oxides such as kaolinite can also occur in the same region.

Krzebietke et al. [14] proposed a method to determine iron and other metals in soils using NIR spectroscopy with PLS regression with detrending as the preprocessing algorithm. The RMSEP values were comparable in the iron range concentration. The concentration range of iron [14] was 0.70–4.00 × 10^3^ mg/kg. In their article, the range was 9.3–69.0 × 10^3^ mg/kg. In terms of number of latent variables, Krebietke et al. obtained 9 versus 12 and an R^2^ of 0.76 versus 0.79 compared to our results.

Maia et al. [19], determining iron in soil using NIR spectrometry, obtained the best chemometric model using random forest as the regression algorithm and detrending as the preprocessing method. In comparison to Maia et al., the proposed model in our article obtained better RMSEP (4.58 × 10^3^ versus 8.70 × 10^3^ mg/kg), RPD (2.21 versus 1.36) and R^2^ (0.79 versus 0.50) using 1350 versus 2500 variables [19].

Naibo et al. [37] analyzed Fe and obtained a better result for a RMSEP of 2.90 × 10^3^ mg/kg using full spectra with the Savitzky–Golay derivative as the preprocessing method in NIR data; their R^2^ equal to 0.99 indicated, however, an overfitting method. The authors indicated that their method of Ti determination was not accurate.

Mammadov et al. [40] determined Mehlich 3 extractable elements including iron using visible and NIR spectral regions, PLS regression and Savitzky–Golay preprocessing using first derivative with a gap segment size of 10 bands. The R^2^ of calibration and prediction (0.83 versus 0.82 and 0.76 versus 0.79, respectively) were comparable with our study and the RPD obtained in their work was better (2.21 versus 1.72).

### 2.3. Determination of Aluminum, Beryllium, Gadolinium and Yttrium

For Al, Be, Gd and Y, only the FFiPLS algorithm (Table 4) presented the EJCR at a specific point on the ellipse of confidence for the calibration and prediction models, using a smaller number of latent variables. Values for RMSECV, RMSEP, bias_pred_ and SDV obtained for Be, Gd and Y were lower than for Al. This can be explained by the higher Al concentration in the sample set (47.1–157.8 × 10^3^ mg kg^−1^).

**Figure 4:**
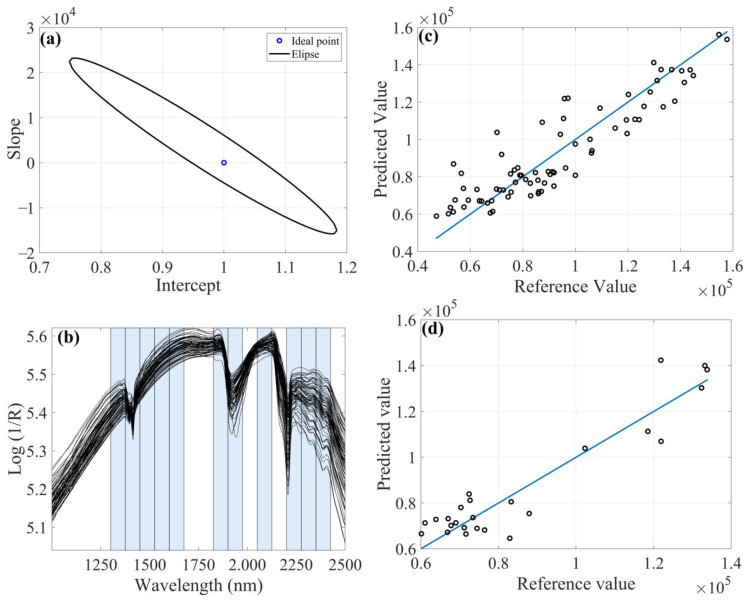
FFiPLS chemometric model with MSC as preprocessing for determination of aluminum content: (**a**) EJCR; (**b**) Selected spectral regions; (**c**) Predicted versus reference values for calibration sample set; (**d**) Predicted versus reference values for prediction sample set.

For Al determination, the preprocessed model using MSC (Figure 4) showed lower REP (10.20%), RMSEP (8.80 × 10^3^ mg kg^−1^) and SDV (9.09 × 10^3^ mg kg^−1^) values, as well as higher linearity due to R^2^_pred_. In some cases, it is important to evaluate the viability of the model not only by the highest R^2^ value, since this parameter only indicates the variance explained by the linear equation.

Maia et al. [19], determining aluminum in soil using NIR spectrometry, obtained the best chemometric model using PLS as the regression algorithm and SNV as the preprocessing method. Compared with this result, the proposed model in our article obtained better RMSEP (8.80 × 10^3^ versus 11.8 × 10^3^ mg/kg), RPD (2.79 versus 2.12) and R^2^ (0.87 versus 0.76) [19].

Naibo et al. [37] analyzed aluminum and obtained a better result, with a RMSEP of 1.47 × 10^3^ mg/kg using full spectra with the Savitzky–Golay derivative as the preprocessing method with NIR data, but the R^2^ equal to 0.99 indicated an overfitting method.

Gholizadeh et al. [41] proposed a method to determine aluminum in forest soils using visible-NIR spectroscopy and learning algorithms. The best model in the work obtained an R^2^ equal to 0.86 and RMSEP of 1.50 × 10^3^ mg/kg, comparable to our study, considering the difference between concentration ranges (0.31–29.3 × 10^3^ versus 47.1–157.8 × 10^3^ mg/kg).

When the RMSEP is not low enough, it is interesting to know the bias to evaluate the technique used. High values in the bias_pred_ indicate low veracity in the measurement; therefore, the model obtained for the raw data matrix is not ideal, despite the F-Test showing that statistically there are no significant differences between them.

The spectral range 1375–1450 nm can be assigned the vibrational frequencies of -OH groups in the adsorbed water by the vibrational combinations of the metal with hydroxyl (Al-OH) plus O-H stretching. The spectral region 2200–2275 nm may be associated with the combination of Al-OH plus O-H stretching bend vibrations in poorly ordered kaolinite (near to 2205 nm) and Al-OH from 2:1 clay minerals (2160 nm). In the literature, reflectance spectral characteristics of clay minerals are reported, which indicates that the spectrum of kaolinite is characterized by a strong hydroxyl absorption band with aluminum coordination and aluminum oxides (Al_2_O_3_).

**Figure 5:**
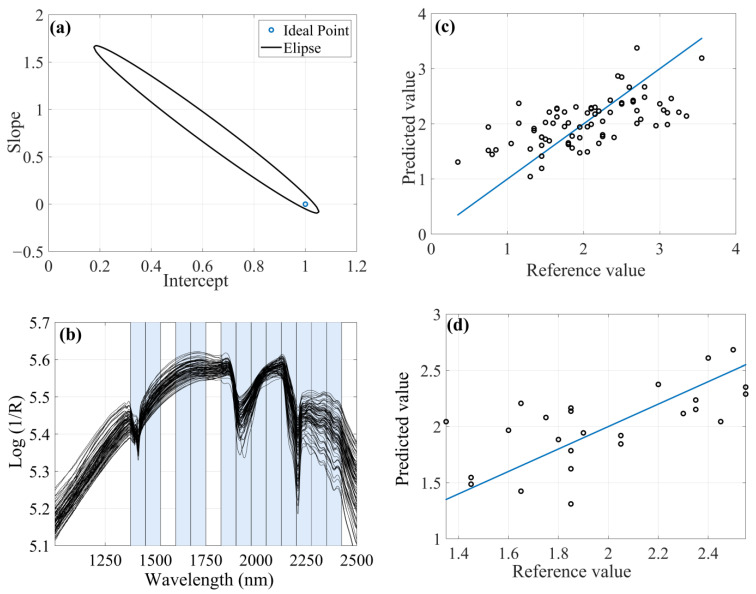
FFiPLS chemometric model with MSC as preprocessing for determination of beryllium content: (**a**) EJCR; (**b**) Predicted versus reference values for calibration sample set; (**c**) Selected spectral regions; (**d**) Predicted versus reference values for prediction sample set.

For Be (0.35 to 3.55 mg kg^−1^), as shown in Figure 5, the model employing the FFiPLS algorithm preprocessed by MSC was shown to be superior as it presented lower values of RMSEP (0.29 mg kg^−1^), REP (14.81%), SDV (0.30 mg kg^−1^) and LV (4) as well as higher linearity (R^2^_pred_ = 0.3354). Naibo et al. [37] obtained a RMSEP of 0.13 mg/kg using full spectra with Savitzky–Golay derivative as the preprocessing method in NIR data, but the R^2^ equal to 0.99 indicated an overfitting method.

According to the literature, metals at low concentrations are not spectrally active in the NIR region because their signals may be overlapped by more intense signals where they are embedded in clay mineral structures or associated with organic matter. This may explain why some models proved unreliable by not showing the optimum within the ellipse point in the EJCR test.

**Figure 6:**
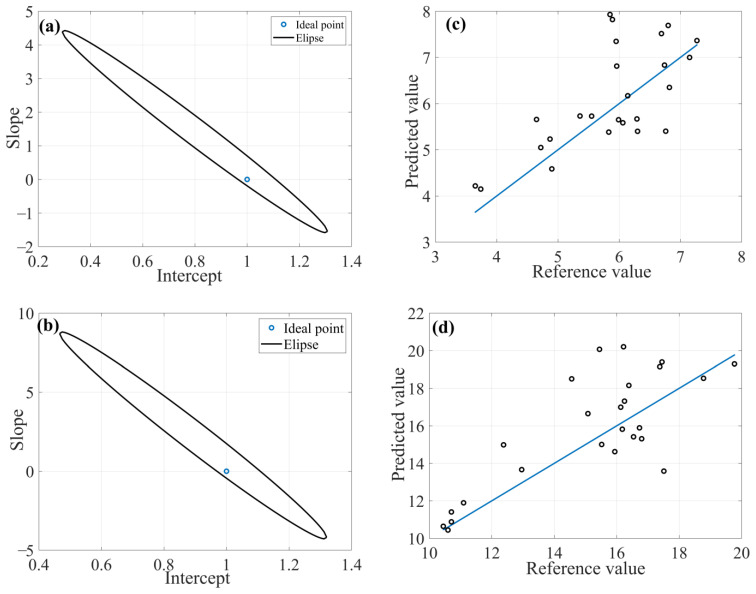
FFiPLS chemometric model with SG smoothing as preprocessing for determination of gadolinium and yttrium contents: (**a**) EJCR from Gd; (**b**) EJCR from Y; (**c**) Predicted versus reference values for prediction set from Gd; (**d**) Predicted versus reference values for prediction set from Y.

Gd and Y are indispensable for high-tech production (computers, wind towers, light-emitting diodes and others). For both, only FFiPLS (Figure 6) provided the best results with the Savitzky–Golay smoothing preprocessing, but the quality model was not accurate. For Gd and Y, the EJCR showed a point within the confidence ellipse where the deviation of the samples was low. Also, lower SDV, bias_pred_, RMSECV and RMSEP values were observed. This was probably because the working ranges of Gd and Y are lower than those of Al and therefore influence the determination coefficients.

Maia et al. [19] published an article that determined Be using PLS with continuum removal as the preprocessing algorithm; their results were comparable with our study in terms of R^2^ (0.20 versus 0.35), RMSE (0.85 versus 3.47 mg/kg), bias (0.25 versus 0.96 mg/kg) and RPD (1.12 versus 1.23).

## 3. Materials and Methods

### 3.1. Study Area

Soil samples were selected from the Ipojuca River watershed located in the state of Pernambuco, between parallels 8° 09′ 50″ and 8° 40′ 20″ south latitude and meridians 34° 57′ 52″ and 37° 02′ 48″ longitude west of Greenwich. The basin has a strategic position, linking the Metropolitan Region of Recife and the backwoods regions of state. The river area covers a surface of 3433.58 km^2^ corresponding to 3.49% of the total state and perimeter of 749.6 km. Most of the area of the Ipojuca River basin is represented by crystalline rocks from the Precambrian era. The dominant lithostratigraphic is the Migmatitic–Granitoid Complex, where granites and granodiorites are predominant over migmatites. Small areas also are associated with metagraywacke quartzites and crystalline limestones, besides schists and undifferentiated gneisses.

### 3.2. Soil Analysis and Parameters of Interest

A total of 101 soil samples (0–5 cm depth) were collected along the river basin. The soil samples were air dried in an oven at 50 °C for 48 h. They were disaggregated and sifted through a 2 mm mesh and finally separated by sifting at ≤100 μm.

The concentrations of different metals from the 101 samples were measured by inductively coupled plasma optical emission spectrometry (ICP-OES) using an Optima DV7000 spectrophotometer, PerkinElmer. The metals determined were aluminum, beryllium, iron, titanium, gadolinium and yttrium. The measurements were performed after extraction by acid digestion on a heating plate (~180 °C) employing hydrofluoric (10 mL), nitric (5 mL) and perchloric (3 mL) acids following the proposed methodology [42]. The extracts were dissolved in hydrochloric acid and diluted in deionized water.

### 3.3. Spectral Analysis and Database

After drying in an oven at 50 °C for 48 h, the samples were measured in the FT-NIR spectrometer, PerkinElmer, with a reflectance accessory. The NIR spectra were obtained between 1000 and 2500 nm with 2 nm resolution and 32 independent scans for sample at wavelength steps of 0.5 nm. The dataset included 101 observations (samples) with 3001 wavelengths (variables).

### 3.4. Chemometric Methods

The chemometric models were built with raw data and the following preprocessing of the data (spectra): multiplicative scatter correction (MSC); standard normal variate (SNV); mean centering; adjustment of baseline; smoothing and derivation by the Savitzky–Golay method (using 1st derivate, 2^nd^-degree polynomial and 17-point window); mean reduction; and smoothing by the moving average method. This preprocessing is a crucial step to build calibration models using NIR as the analytical technique [43] to remove unwanted or harmful signals. The main problems in NIR spectroscopy are baseline shift, vertical offsets, spurious scattering of radiation and spectral noises.

The samples were divided into calibration (76) and prediction (25) sets for each preprocessed dataset using the SPXy algorithm from the Data Hand Gui interface [44], in Matlab^®^ version R2016a. The samples of calibration sets were used to build the chemometric models and prediction sets to evaluate the built models.

The algorithms used to build the chemometric models were PLSR, iPLS [45] and iSPA-PLS using iSPA Gui interface [46] and FFiPLS. The number of latent variables for each PLS model was selected using the root mean square error of the cross-validation (RMSECV). The iPLS, iSPA-PLS and FFiPLS models were built by dividing the spectra into 20 intervals. The parameters used in the FFiPLS algorithm were 50 Fireflies (ffpop), 50 cycles (generations) and the values attributed to w_0_, gamma (γ) and alpha (α), respectively, 0.97, 1.0 and 0.2. All algorithms were carried out using Matlab^®^ version R2016a.

The results were evaluated and chemometric models compared using the predictive ability in terms of RMSEC, R^2^_cal_, R^2^_pred_, bias_pred_, RPD, SDV and REP [47].

## 4. Conclusions

Through this study, it was possible to build models for prediction of different metals (aluminum, beryllium, iron, titanium, gadolinium and yttrium) using a set of soil samples from deterministic (PLS, iPLS, iSPA-PLS) and stochastic (FFiPLS) variable-selection techniques. The FFiPLS algorithm provided more appropriate results for some analytes, employing fewer latent variables and achieving lower values of RMSEP, RMSECV, REP, SDV and bias_pred_.

FFiPLS outperformed the deterministic iPLS, iSPA-PLS and full PLSR algorithms for the determination of Al, Fe and Ti based on their high presence in the soil samples. Although the deterministic algorithms expressed solutions with good performance, as the number of variables increased, they started to fail. This could be seen in the case of Be, Gd and Y; due to the very low concentration of metals, however, the results were not satisfactory for metals. The raw matrix data did not provide significant results, probably due to a number of properties that influenced the soil, such as moisture, organic matter and particle size. Thus, different preprocessing techniques were employed on the reflectance database obtained by NIR spectroscopy. This procedure was crucial for building the calibration models using NIR as the analytical technique. Thus, the preprocessing techniques used in this article were Savitzky–Golay, derivations, MSC and SNV.

The determination of metals in soil is important in order to determine the type and agronomic conditions of soils and for other exploratory activities of soils such as extraction of metals. But the analytical process to determine these analytes uses expensive reagents and instruments, and qualified labor, and it demands significant time. Thus, methods that use NIR spectroscopy with chemometric tools associated with variable selection, such as FFiPLS, are an interesting alternative for determining metals in soils in an economic, rapid and precise manner.

## Figures and Tables

**Table 1:** Basic statistics concerning to selected metals determination in mg kg^−1^.

Elements	Minimum Value	Maximum Value	Mean Value	SD	RSD
Ti	1.60 × 10^3^	10.4 × 10^3^	4.66 × 10^3^	2.01 × 10^3^	43.08
Fe	9.3 × 10^3^	69.0 × 10^3^	30.6 × 10^3^	13.0 × 10^3^	42.41
Al	47.1 × 10^3^	157.8 × 10^3^	91.2 × 10^3^	27.7 × 10^3^	30.42
Be	0.35	3.55	2.02	0.62	30.69
Gd	2.44	15.24	5.60	1.62	28.97
Y	6.82	35.80	14.77	4.03	27.29

SD—Standard Deviation; RSD—Relative Standard Deviation.

**Table 2:** Figures of merit of chemometric models for titanium content determination.

Preprocessing	MSC		SNV	BaseLine (Linear)	BaseLine (OffSet)	BaseLine (Off + Linear)
Model	iSPA-PLS	FFiPLS	FFiPLS	FFiPLS	PLS	FFiPLS
LV	21	10	11	6	17	12
NV	2550	750	1500	1350	3001	1350
RMSEC (mg kg^−1^)	0.36 × 10^3^	0.92 × 10^3^	0.83 × 10^3^	1.02 × 10^3^	0.61 × 10^3^	0.80 × 10^3^
R^2^_cal_	0.9792	0.8381	0.8743	0.7876	0.9353	0.8802
RMSEP (mg kg^−1^)	0.73 × 10^3^	0.62 × 10^3^	0.87 × 10^3^	0.77 × 10^3^	0.79 × 10^3^	0.80 × 10^3^
R^2^_pred_	0.7097	0.7862	0.5655	0.6725	0.6881	0.7055
Bias_pred_ (mg kg^−1^)	0.28 × 10^3^	0.27 × 10^3^	0.34 × 10^3^	0.19 × 10^3^	0.05 × 10^3^	0.16 × 10^3^
REP (%)	18.19	15.6	21.25	19.76	19.99	19.96
RPD_pred_	1.85	2.16	1.52	1.75	1.79	1.84
SDV	0.89 × 10^3^	0.79 × 10^3^	1.07 × 10^3^	0.85 × 10^3^	0.81 × 10^3^	0.86 × 10^3^

LV—Number of Latent Variables; NV—Number of Variables; RMSEC—Root Mean Square Error of Calibration; RMSEP—Root Mean Square Error of Prediction; REP—Relative Error of Prediction; RPD—Residual Prediction Deviation; SDV—Standard Deviation of Validation.

**Table 3:** Figures of merit of chemometric models for determination of iron content.

Preprocessing	SNV		Moving (Average)	Baseline (Linear)		Baseline (Offset)	Baseline (Offset + Linear)
Model	iPLS	FFiPLS	PLS	iSPAPLS	iPLS	FFiPLS	FFiPLS
LV	13	12	16	13	11	11	14
NV	150	1350	3001	150	150	750	150
RMSEC (mg kg^−1^)	2.16 × 10^3^	6.23 × 10^3^	4.68 × 10^3^	1.63 × 10^3^	3.41 × 10^3^	6.38 × 10^3^	1.00 × 10^3^
R^2^_cal_	0.9791	0.8237	0.9099	0.9882	0.9472	0.8152	0.9958
RMSEP (mg kg^−1^)	5.81 × 10^3^	4.58 × 10^3^	8.09 × 10^3^	6.21 × 10^3^	5.88 × 10^3^	6.22 × 10^3^	6.16 × 10^3^
R^2^_pred_	0.6701	0.7947	0.0135	0.4910	0.5439	0.4888	0.4464
Bias_pred_ (mg kg^−1^)	0.46 × 10^3^	0.46 × 10^3^	1.70 × 10^3^	0.37 × 10^3^	0.60 × 10^3^	0.52 × 10^3^	1.53 × 10^3^
REP (%)	21.61	17.04	31.78	24.42	23.12	24.47	23.26
RPD_pred_	1.74	2.21	1.01	1.4	1.48	1.4	1.34
SDV	5.91 × 10^3^	4.65 × 10^3^	8.79 × 10^3^	6.37 × 10^3^	6.09 × 10^3^	6.33 × 10^3^	6.09 × 10^3^

LV—Number of Latent Variables; NV—Number of Variables; RMSEC—Root Mean Square Error of Calibration; RMSEP—Root Mean Square Error of Prediction; REP—Relative Error of Prediction; RPD—Residual Prediction Deviation; SDV—Standard Deviation of Validation.

**Table 4:** Figures of merit of chemometric models for aluminum, beryllium, gadolinium and yttrium content determinations.

Analyte	Al				Be		Gd	Y
Preprocessing	Raw Data	SNV	MSC	Baseline (Offset + Linear)	MSC	SNV	SG Smoothing	SG Smoothing
LV	9	7	7	6	4	5	5	5
NV	1200	1800	1650	1350	1800	750	1650	1050
RMSEC (mg kg^−1^)	12.77 × 10^3^	13.09 × 10^3^	12.82 × 10^3^	13.31 × 10^3^	0.55	0.55	1.40	3.37
R^2^_cal_	0.8203	0.8048	0.8165	0.8008	0.3812	0.4059	0.4276	0.4489
RMSEP (mg kg^−1^)	12.16 × 10^3^	11.61 × 10^3^	8.80 × 10^3^	9.50 × 10^3^	0.29	0.34	0.85	1.98
R^2^_pred_	0.7729	0.8023	0.872	0.8533	0.3354	0.0488	0.2029	0.4437
Bias_pred_ (mg kg^−1^)	3.89 × 10^3^	0.25 × 10^3^	0.79 × 10^3^	0.72 × 10^3^	0.02	0.02	0.25	0.65
REP (%)	14.06	13.01	10.2	10.65	14.81	17.19	14.55	13.09
RPD_pred_	2.1	2.25	2.79	2.61	1.23	1.02	1.12	1.34
SDV	14.19 × 10^3^	11.85 × 10^3^	9.09 × 10^3^	9.67 × 10^3^	0.3	0.35	0.98	2.32

LV—Number of Latent Variables; NV—Number of Variables; RMSEC—Root Mean Square Error of Calibration; RMSEP—Root Mean Square Error of Prediction; REP—Relative Error of Prediction; RPD—Residual Prediction Deviation; SDV—Standard Deviation of Validation.

## Data Availability

The data presented in this study are available on request from the corresponding author.

## Sample Availability

Not available.
